# Posttranslational Modifications in PD-L1 Turnover and Function: From Cradle to Grave

**DOI:** 10.3390/biomedicines9111702

**Published:** 2021-11-16

**Authors:** Xinfang Yu, Wei Li, Ken H. Young, Yong Li

**Affiliations:** 1 Section of Epidemiology and Population Science, Department of Medicine, Baylor College of Medicine, One Baylor Plaza, Houston, TX 77030, USA; xinfang.yu@bcm.edu (X.Y.); liweilx@gmail.com (W.L.); 2 Hematopathology Division, Department of Pathology, Duke University Medical Center, Durham, NC 27710, USA; ken.young@duke.edu

**Keywords:** programmed death-ligand 1, programmed death 1, posttranslational modifications, stability, translocation, immunotherapy

## Abstract

Programmed death-ligand 1 (PD-L1) is one of the most classic immune checkpoint molecules. Cancer cells express PD-L1 to inhibit the activity of effector T cells’ cytotoxicity through programmed death 1 (PD-1) engagement in exposure to inflammatory cytokines. PD-L1 expression levels on cancer cells might affect the clinical response to anti-PD-1/PD-L1 therapies. Hence, understanding molecular mechanisms for regulating PD-L1 expression is essential for improving the clinical response rate and efficacy of PD-1/PD-L1 blockade. Posttranslational modifications (PTMs), including phosphorylation, glycosylation, ubiquitination, and acetylation, regulate PD-L1 stability, cellular translocation, and interaction with its receptor. A coordinated positive and negative regulation via PTMs is required to ensure the balance and function of the PD-L1 protein. In this review, we primarily focus on the roles of PTMs in PD-L1 expression, trafficking, and antitumor immune response. We also discuss the implication of PTMs in anti-PD-1/PD-L1 therapies.

## 1. Introduction

Immunotherapies such as T cell adoptive transfer, mRNA vaccines, and checkpoint inhibitors are effective cancer treatment strategies [1]. Monoclonal antibodies against programmed death 1 (PD-1) or its ligand, programmed death-ligand 1 (PD-L1) [2], have opened a new era for cancer therapy [3,4,5,6,7]. PD-L1 (also known as CD274 or B7-H1) is highly expressed in various types of cancers, including melanoma, lymphoma, lung cancer, bladder cancer, and kidney cancer [8,9]. Elevated PD-L1 on cancer cells engages PD-1 on T cells, leading to T cell dysfunction and exhaustion and preventing cytotoxic T cells from effectively killing the cancer cells. Based on the favorable therapeutic outcomes from anti-PD-1/PD-L1 therapy, PD-L1 has become a key protein in immuno-oncology, and its functions and regulatory mechanisms are intensively studied. There are multi-level mechanisms to regulate PD-L1 protein tightly, including (1) genetic alterations and epigenetic modifiers such as gene amplification, translocation, and 3′-UTR disruption; (2) transcriptional regulation such as transcriptional factors activity or upstream signaling pathways; and (3) posttranslational modifications (PTMs) of proteins [7,9,10]. Our understanding of the regulation of PD-L1 will help improve the efficacy of immune checkpoint blockade and will advance cancer immunotherapy. In this work, we review the research progress of PD-L1 PTMs in regulating their expression and function.

Proteins are synthesized by ribosomes, translating mRNA into polypeptide chains before undergoing PTMs to produce mature protein molecules. PTMs are covalent additions of functional groups such as phosphate, methyl, ubiquitin, and acetate to the protein substrates by different enzymes [11]. PTMs include phosphorylation, glycosylation, ubiquitination, methylation, and acetylation, playing essential roles in regulating protein activity, stability, translocation, and protein–protein interactions [11,12]. Nearly all protein synthesis and PTMs occur in the cytosol, where a complex system of targeting, sorting, recycling, and consigning is in place. With such a system, newly synthesized proteins are localized to their correct cellular compartments. As a membrane protein, PD-L1 has to be exposed at the cell surface, where it binds to PD-1 and activates downstream effectors [2]. Because immunotherapy blocks the PD1–PD-L1 interaction at the cell surface, the recycling and relocalization of intracellular PD1 and PD-L1 may impact the efficacy of immunotherapy to a large extent. Recent studies have attempted to reduce the PD-L1 expression intrinsically by interfering with its regulators [9]. The PD-L1 protein must be organized and tightly controlled spatially and temporally within the cell to function appropriately. The organization and control are ensured by intracellular machinery and rely on membrane trafficking events in cells that are often guided by various PTMs. Horita and colleagues first reported that PD-L1 is subjected to acetylation, tyrosine phosphorylation, and mono-ubiquitination upon epidermal growth factor (EGF) stimulation [13]. Moreover, a significant increase in mono- and multi-ubiquitination of PD-L1 occurred on glycosylated PD-L1 [13]. The increased PD-L1 mono- and multi-ubiquitination were blocked by gefitinib treatment [13]. This study opens the door to identifying novel PTMs for PD-L1 and reveals potentially critical regulatory mechanisms that may be valuable therapeutic targets. Recently, increasing evidence demonstrates that PD-L1 PTMs affect their stability, distribution, and interaction with PD-1 in regulating immunosuppression [14].

**Figure 1:**
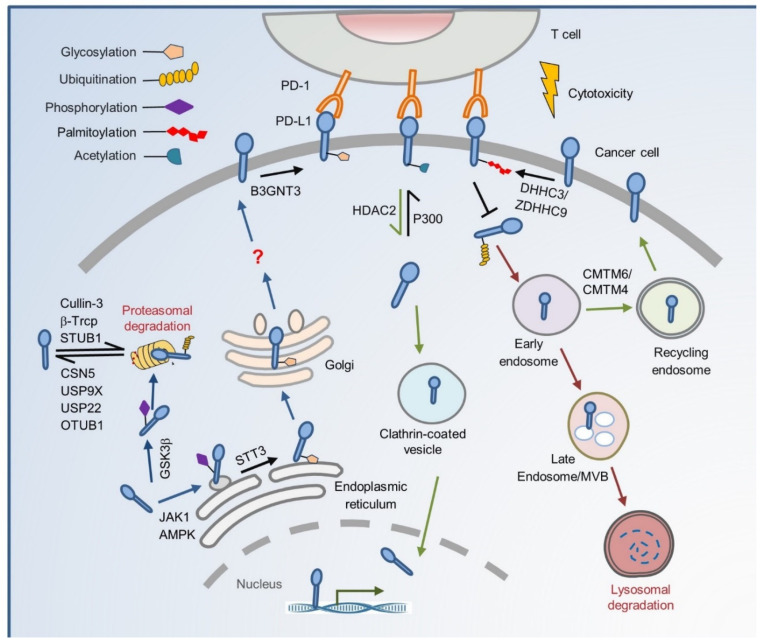
The regulation of PTMs on subcellular transportation of PD-L1 in cancer cells. As a membrane protein, PD-L1 is modified by many PTMs after its translation. *N*-glycosylation of the PD-L1 extracellular domain occurs in the lumen of ER, and this modification, mediated by STT3A, requires JAK1-mediated PD-L1 phosphorylation. Glycosylation also inhibits phosphorylation by GSK3β, thereby blocking the ubiquitination by β-TrCP. AMPK phosphorylates PD-L1 to induce abnormal ER mannose trimming during PD-L1 glycosylation. The abnormal glycosylation of PD-L1 accumulates in the ER and is no longer transported to the Golgi. Other E3 ligases such as Cullin3 and STUB1 also degrade PD-L1 by proteolysis. Deubiquitination by CSN5, USP9X, USP22, and OTUB1 protects PD-L1 from proteasomal degradation. B3GNT3-mediates PD-L1 glycosylation and helps it interact with PD-1 on the cell membrane. PD-L1 may be transported from cell membrane to lysosome for destruction or recycling through a series of endosome trafficking. Palmitoylation by DHHC3/ZDHHC9 blocks PD-L1 ubiquitination, thereby preventing its internalization to the MVB and lysosome degradation. CMTM6/CMTM4 binds PD-L1 and maintains its cell surface expression through recycling endosomes but not lysosomal degradation. This process may stabilize PD-L1 by suppressing its ubiquitination. PD-L1 deacetylated by HDAC2 is translocated from the plasma membrane into the nucleus through clathrin-mediated endocytosis. Unacetylated PD-L1 interacts with HIP1R and cargo proteins leading to nuclear translocation through the cytoskeleton and then transactivates immune responsive genes to impact the PD-1/PD-L1 blockage treatment response.

Characterization of the functional impacts of PTMs on PD-L1 will extend our understanding of the regulatory network behind the PD-L1 protein level and provide new approaches to improve immunotherapy efficacy. This review summarizes recent PTMs on PD-L1 protein regulation from cradle to grave and their potential therapeutic roles in cancer treatment (Figure 1).

## 2. The Cradle: From De Novo Synthesis to Plasma Membrane

In general, de novo synthesized proteins that are folded correctly are packaged into coat protein complex II (COPII) vesicles and transported from the endoplasmic reticulum (ER) to the Golgi complex [15]. The ER serves as the protein quality control center. Proteins are folded and posttranslationally modified as they traffic from the ER to the Golgi complex and trans-Golgi network (TGN), then transported to the plasma membrane or other organelles by vesicular carriers [16]. However, lumenal or integral membrane proteins that do not fold correctly are retained in the ER and are subject to ER-associated degradation (ERAD) [17]. The ERAD provides a crucial mechanism for proteins exiting the ER, especially for membrane protein degradation, to limit their surface expression. This elaborate process is initiated by substrate recognition, which includes prolonged association with ER chaperones or modified glycan processing. PD-L1 is a transmembrane domain (TMD) protein [18]. After PTMs, newly synthesized PD-L1 protein is transported to the cell surface through the ER–Golgi intermediate compartment trafficking pathway. PD-L1 transportation to the cell surface is essential for maintaining its homeostasis to generate an immunosuppressive effect. Once the PTMs-regulated intracellular trafficking becomes chaotic, cellular dysfunction and subsequent disorder occur.

Specific glycosylation is critical for membrane proteins’ intracellular transport from one cargo to the next [19]. It is essential for many glycoproteins to be sorted into transport containers in the trans-Golgi network and/or endosomes, followed by their delivery to the appropriate plasma membrane domains [20]. The N-linked glycosylation process is the attachment of an oligosaccharide, a carbohydrate consisting of several sugar molecules, sometimes referred to as glycan, to a nitrogen atom (the amide nitrogen of asparagine (N) residue of a protein) [21]. In this process, initial trimming of the precursor molecule occurs in the ER by oligosaccharyltransferase, which transfers a 14-sugar core glycan from dolichol to an asparagine residue of an N-X-T/S motif (asparagine-any amino acid except proline-threonine/serine) in newly synthesized nascent proteins that have entered the ER lumen [22]. The core glycan is then trimmed and further processed in the Golgi apparatus before the glycosylated protein is translocated to the cell membrane [23]. When glycosylation is dysregulated, the protein is transported to the cytosol and rapidly undergoes ERAD. Moreover, other PTMs such as phosphorylation and ubiquitination work interdependently to make the regulation process more accurate.

It has been reported that N-linked glycosylation happens on PD-L1 [24]. Glycosylated PD-L1 has a half-life of ~12 h, while non-glycosylated PD-L1 undergoes rapid proteolysis, with a half-life of ~4 h. Two co-chaperones, Sigma1 [25] and FKBP51 [26], interact with and help glycosylated PD-L1 to fold correctly and stabilize in the ER lumen, and then transport it to the membrane via Golgi through the secretory pathway. If glycosylation is dysregulated, PD-L1 will undergo ubiquitination and subsequent degradation by the proteasome. Four N-X-T/S motifs of PD-L1 (N35, N192, N200, and N219) are found with N-linked glycosylation, and three (N192, N200, and N219) of them contribute to PD-L1 protein stability [24]. Glycosylation on these three asparagine sites antagonizes their interaction with glycogen synthase kinase 3 beta (GSK3β) [24]. In contrast, non-glycosylated PD-L1 binds to and is phosphorylated by GSK3β at T180 and S184. Phosphorylation of PD-L1 induces its association with the E3 ligase beta-transducing repeats-containing protein (β-TrCP), which results in PD-L1 degradation [24]. A key component responsible for PD-L1 *N*-glycosylation is the catalytic subunit of oligosaccharyltransferase STT3A [27], which transfers the core glycan structure to PD-L1, resulting in PD-L1 protein *N*-glycosylation and stabilization. Another study revealed that PD-L1 association with STT3A in ER requires JAK1-mediated PD-L1 phosphorylation at tyrosine112 (Y112), which results in glycosylation of PD-L1 and trafficking to the cell surface [28]. AMP-activated protein kinase (AMPK) phosphorylates PD-L1 at S195 to induce abnormal ER mannose trimming during PD-L1 glycosylation [29]. The abnormally glycosylated PD-L1 is no longer transported to the Golgi. Instead, it accumulates in the ER and is subsequently degraded via ERAD. In addition to polyubiquitination, EGF treatment induces the mono- and multi-ubiquitination of glycosylated PD-L1 to maintain its stability [13]. However, the specific E3 ligase for promoting the mono-ubiquitination of PD-L1 remains elusive. With or without glycosylation, ubiquitination of PD-L1 mediated by the E3 ligase that consists of Cullin-3 and the adaptor protein speckle-type POZ protein (SPOP) degrades PD-L1 protein in late G1 and S phases [30].

Deubiquitination is a reversible process of ubiquitination where deubiquitinating enzymes (DUBs) remove ubiquitin (ub), ub-like molecules, or remodel ub-chains from the target proteins [31]. COP9 signalosome 5 (CSN5) was the first identified DUB to inhibit the ubiquitination and degradation of PD-L1 [32]. The stabilization of PD-L1 results in tumor necrosis factor-alpha (TNF-α)-triggered cancer cell immune escape from T cell surveillance [32]. Recently, more DUBs, such as ubiquitin-specific peptidase 9 X-linked (USP9X) [33], USP22 [34,35], and OTU domain ubiquitin aldehyde binding 1 (OTUB1) [36], were found to deubiquitinate and stabilize PD-L1 in different cancers. These DUBs interact with PD-L1 and remove the K48-linked ubiquitin chain from PD-L1 to hinder its degradation through the ERAD pathway.

**Figure 2:**
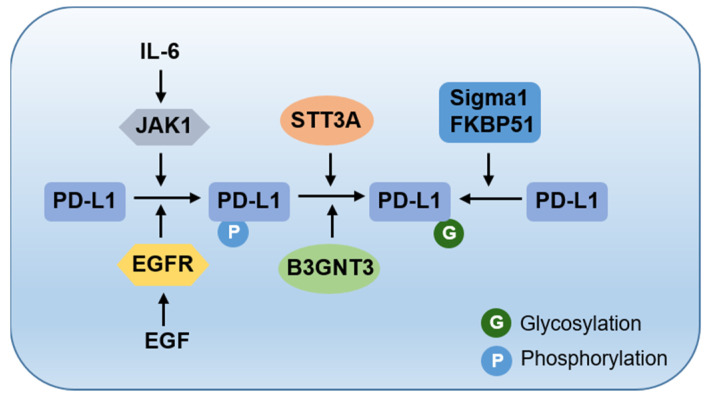
The crosstalk between phosphorylation and glycosylation. JAK1- and EGFR-mediated phosphorylation serves as a signal for the addition of glycosylation on PD-L1.

When adequately folded PD-L1 arrives at the cell surface, glycosylation is involved in the physical interaction between PD-L1 and PD-1 and exerts an immunosuppressive function. The glycosylated PD-L1 engages with PD-1, whereas its non-glycosylated mutant fails to do so [37]. Bioinformatic analysis and biochemical experiments have shown that β1,3-*N*-acetylglucosaminyltransferase 3 (B3GNT3)-mediated poly-*N*-acetyllactosamine (poly-LacNAc) regulates PD-L1 glycosylation [37]. Unlike the stabilization-related sites, the two asparagine sites (N192 and N200) of PD-L1 glycosylation are required for the PD-L1–PD-1 interaction. N-linked glycans attach to a nitrogen of asparagine or arginine side-chains. O-linked glycans attach to the hydroxyl oxygen of serine, threonine, tyrosine, hydroxylysine, or hydroxyproline side-chains, or to oxygens on lipids such as sphingosine of ceramides. The interaction can be blocked by N-linked glycosylation inhibitors, but not by O-linked glycosylation inhibitors [37]. Thus, PD-L1 glycosylation on different asparagine sites mediated by enzymes STT3A and B3GNT3 plays a number of distinct functions, leading to PD-L1 degradation or interaction with PD-1. Furthermore, there is a positive crosstalk between different PTMs, where phosphorylation serves as a signal for the addition of glycosylation (Figure 2). Understanding these detailed processes would improve the efficiency of structure-based drug design targeting this crucial immune suppression signaling pathway.

In addition to its intracellular distribution, PD-L1 has also been detected outside the cell. Recent studies report that exosomal PD-L1 isolated by high-speed centrifugation is an essential form of PD-L1 and systemically inhibits an antitumor immune response [38,39,40,41,42]. Moreover, an increased level of soluble PD-L1 exists in plasma samples and correlates with poor prognosis in cancer patients [43,44,45,46,47,48,49]. Soluble PD-L1 protein levels outside the cell may serve as a predictive biomarker for cancer prognosis and individualized immunotherapy. How PTMs affect soluble PD-L1 protein levels and function remains unknown.

## 3. To the Grave: From Cell Membrane to Recycling and Lysosome Degradation

Plasma membrane proteins are often removed from the cell surface to keep the plasma membrane composition balance. The canonical method by which a cell removes membrane proteins is via endocytosis, where the plasma membrane and its integral membrane proteins bud inward and are transported to the endosomes [50]. Endocytosis occurs via clathrin-dependent and -independent mechanisms [51]. Upon internalizing the proteins to intracellular trafficking, cargos are transported to the early endosomes, where proteins can be redistributed to their appropriate cellular location. Proteins in early endosomes can be recycled to the plasma membrane (with or without passing through the recycling endosome), transferred to the late endosomes before moving to the lysosome for degradation, or subjected to retro-translocation to the trans-Golgi, nuclear, and other cell organelles. Endocytosis and recycling contribute to cell surface proteins homeostasis.

PD-L1 plays an immunosuppression function on the plasma membrane surface, and it may translocate into the cytoplasm after functioning. Endocytosis shuttles membrane PD-L1 molecules between the cell surface and cytoplasm through endosomes. Two independent studies have identified the CKLF-like MARVEL transmembrane domain-containing protein 6 (CMTM6) as the critical positive regulator for PD-L1 transportation into recycling endosomes. Using a genome-wide CRISPR-Cas9 screen, one group showed that CMTM6 binds and colocalizes with PD-L1 at the plasma membrane and in early endosomes [52]. CMTM6-PD-L1 is transferred to the recycling endosome, which helps endocytosed PD-L1 recycle to the cell surface, preventing PD-L1 from lysosome-mediated degradation and increasing its protein pool [52]. This study did not examine whether PTMs regulate the degradation–recycle process. Using a haploid genetic screen, another group demonstrated that CMTM6 interacts with PD-L1 at the cell surface, reduces its ubiquitination, and increases the PD-L1 protein half-life [53]. They also identified that CMTM4, but not other CMTM family members, plays a complementary function in CMTM6-deficient cells [53]. STIP1 homology and U-box containing protein 1 (STUB1) is an E3 ligase that causes PD-L1 destabilization through ubiquitination [53]. Ubiquitination prevents PD-L1 from being retransferred to the cell membrane surface and promotes its lysosomal degradation. The transportation between the recycling endosome and lysosome controls the fate of PD-L1 protein, which has therapeutic implications for PD-L1 targeting.

Palmitoylation plays an essential role in regulating the subcellular trafficking of proteins between membrane compartments and modulating protein–protein interactions [54]. Protein palmitoylation is the process by which palmitate, a 16-carbon saturated fatty acid, is attached to cysteine (S-palmitoylation), and less frequently to serine and threonine (O-palmitoylation) residues of proteins, through a reversible thioester linkage [54]. PD-L1 is palmitoylated on cysteine residues. Two palmitoyltransferases, ZDHHC9 in breast cancer [55] and ZDHHC3 (DHHC3) in colorectal cancer [56], attach palmitate to the C272 site of PD-L1. ZDHHC9 palmitoylates PD-L1 to maintain its protein stability and cell surface distribution, protecting cancer cells from the immune surveillance of T cells. Disruption of PD-L1 palmitoylation by site-specific mutation (C272A) of PD-L1 or knock-down of ZDHHC9 reduces PD-L1 cell surface distribution, sensitizes breast cancer cells to T cell killing, and inhibits tumor growth in vivo. PD-L1 palmitoylation by DHHC3 regulates its storage and stabilization. Palmitoylation blocks PD-L1 ubiquitination, and endosomal sorting complexes require its sorting by transport (ESCRT) to the multivesicular body (MVB)/lysosome. Inhibition of PD-L1 palmitoylation with a general palmitoylation inhibitor 2-bromopalmitate (2-BP) or knock-down of DHHC3 decreases PD-1 binding and activates T cell cytotoxicity to promote antitumor immunity in the MC38 tumor model. These findings underscore the role of palmitoylation in PD-L1 protein stability and distribution, which involves the molecular masking of an intrinsic lysosomal sorting signal of PD-L1.

PD-L1 is mainly located on the cell membrane and in the cytoplasm, yet it is also found in the nucleus to a lesser extent. Nuclear PD-L1 is detected in many cancer tissues, including renal cell carcinomas, colorectal cancer, prostate cancer, lung cancer, and hepatocellular carcinomas [57,58,59]. Its expression is significantly correlated with tumor invasion, radioresistance, and overall survival, suggesting that nuclear PD-L1 could be a potential prognostic biomarker in cancer patients [57,60]. Karyopherin β1 (KPNB1) binds and mediates PD-L1 nuclear translocation, promoting non-small cell lung cancer cell proliferation [61]. However, it is unclear how PD-L1 enters the nuclear. Wei et al. report that acetylation of PD-L1 mediates its nucleocytoplasmic translocation [62]. PD-L1 is acetylated at the lysine 263 (K263) site in the cytoplasmic domain by p300 acetyltransferase and is de-acetylated by histone deacetylases 2 (HDAC2) [62]. Un-acetylated PD-L1 enables PD-L1 to interact with Huntingtin-interacting protein 1-related protein (HIP1R) and cargo proteins for clathrin-dependent endocytosis, and then with vimentin to traffic through the cytoskeleton, finally translocating into the nucleus through importin-α1 [62]. Moreover, nuclear PD-L1 binds to DNA and regulates the expression of multiple immune-response-related genes to modulate the antitumor immune response. In addition, the PD-L1 nuclear translocation process is independent of its glycosylation status [62]. Interestingly, another group found that HIP1R physically interacts with PD-L1 and delivers it to the lysosome through a lysosome-sorting signal [63]. Thus, HIP1R interaction with PD-L1 regulates its fate through different mechanisms—acetylation-mediated cytoplasmic-nuclear trafficking for gene transcription, or the delivery to lysosomes for degradation.

## 4. Therapeutic Implications of PD-L1 PTMs

Based on the newly identified regulatory mechanism of PD-L1 PTMs, small molecular compounds and antibodies targeting these genes and pathways may modulate PD-L1 expression and function to improve PD1/PD-L1-based immunotherapy.

As we discussed above, PTMs such as glycosylation, phosphorylation, and ubiquitination control PD-L1 stability and function. EGF receptor (EGFR) is the upstream signal that governs GSK3β-mediated PD-L1 phosphorylation and degradation [24]. Targeting EGFR enhances the efficacy of PD-1 blockade in syngeneic mouse models [64]. As EGFR kinase inhibitors are widely used in cancer treatment, clinical trials with both EGFR inhibitors and with PD-1/PD-L1 blockade provide a promising strategy to enhance immunotherapy efficiency [14]. As AMPK-mediated PD-L1 phosphorylation leads to its abnormal glycosylation and degradation, metformin, an AMPK activator, phosphorylates PD-L1 at S195, decreases the stability and membrane localization of PD-L1, and enhances cytotoxic T lymphocyte activity against cancer cells [29]. Interleukin 6-Janus kinase 1 (IL-6-JAK1) signaling mediates phosphorylation of PD-L1 at Y112, which is essential for PD-L1 glycosylation by STT3A to maintain PD-L1 stability [28]. Neutralization of IL-6 with antibody downregulating PD-L1 expression functionally mimics anti-PD-1/PD-L1 effects. Coexpression of T cell immunoglobulin mucin-3 (Tim-3) and PD-1 on tumor-infiltrating lymphocytes is reported to be an indicator of T cell exhaustion. The combination of IL-6 antibody and Tim-3 antibody has proven to be an effective therapy for liver cancer [28]. Glycosylation of PD-L1 prevents its degradation, which in turn stabilizes PD-L1 and suppresses the anti-PD1/PD-L1 immunotherapy effect [24,27,37]; however, no pertinent clinical studies have yet been undertaken. 2-deoxyglucose (2-DG), acting as glucose analog to decrease PD-L1 glycosylation, blocks PD-L1–PD-1 interaction and promotes cytotoxic T cell-mediated antitumor immunity [37,65]. Targeting the PD-L1-specific E3 ligase or DUB is a daunting task. Some strategies such as inhibition of cyclin-dependent kinase 4/6 (CDK4/6) by palbociclib and inhibition of CSN5 by curcumin or berberine were designed to enhance antitumor immunity [32]. PD-L1 stabilization is balanced by ubiquitination-dependent degradation and lysosome-dependent proteolysis. A synthetic peptide (PD-LYSO) that incorporates the lysosome-sorting signal and the PD-L1-binding sequence of HIP1R depletes PD-L1 protein through lysosomal degradation and enhances T cell-mediated cytotoxicity [63]. Cotton et al. constructed antibody-based proteolysis-targeting chimeras (AbTACs) that can target both PD-L1 and the E3 ligase RNF43 to induce the lysosomal degradation of PD-L1 [66]. Bertozzi et al. developed lysosome-targeting chimeras (LYTACs) that can degrade PD-L1 through lysosomes [67].

Most therapeutic antibodies approved by the Food and Drug Administration (FDA) target the cell membrane surface PD-L1 protein in tumors. Apart from controlling the abundance of cell membrane PD-L1, the mechanisms underlying its transportation and structural modulation may also provide novel strategies for cancer treatment and diagnostic detection. The HDAC2 inhibitor santacruzamate A reduces nuclear PD-L1 accumulation and synergizes with anti-PD-1 antibody treatment in the MC38 murine colon carcinoma model [62]. Moreover, PD-1 antibody treatment increases the nuclear PD-L1 signal, which was attenuated after the combined treatment with the HDAC2 inhibitor [62]. PD-L1 is transported from the cell membrane to the lysosome for destruction or recycling through a series of endosome trafficking. Accordingly, inhibition of PD-L1 palmitoylation with a competitive inhibitor 2-BP and knock-out palmitoyltransferase ZDHHC9/DHHC3 decreases the cell surface membrane PD-L1 level and exhausts the storage of PD-L1 in endosomes, thereby enhancing immune clearance of cancer cells [55,56]. CMTM6/CMTM4 binds to and maintains PD-L1 cell surface expression through the recycling of endosomes but not lysosomal degradation. Therefore, the knock-out of CMTM6/CMTM4 alleviates the suppression of tumor-specific T cell activity and strengthens immunotherapy [52,53].

Interestingly, glycosylation of PD-L1 renders its polypeptide antigens inaccessible to PD-L1 antibodies by affecting the protein structure. Removal of PD-L1 *N*-glycosylation with a recombinant glycosidase, peptide-*N*-glycosidase F, allows accurate detection of PD-L1 protein levels [68]. This may help provide a precise prediction of patients who would benefit the most from anti-PD1/PD-L1 immunotherapy. This review summarizes key molecules for PD-L1 PTMs and the mechanisms by which PTMs regulate PD-L1 protein turnover and function (Table 1).

## 5. Future Perspectives

Although targeting PD-1/PD-L1 therapy represents a breakthrough in cancer treatment, its effects are limited to a portion of patients, and resistance often occurs. Therefore, it is necessary to understand the multifaceted regulation of PD-L1 in cancer to enhance the efficacy and response rate of PD-1/PD-L1 blockade. PTMs regulate PD-L1 protein biosynthesis, localization, and functional interaction with other molecules. Membrane PD-L1 protein binds to PD-1 to negatively regulate T cell function, which is a focal point in cancer immunotherapy.

PD-L1 is not only on the cell surface, but it is also distributed in the ER, Golgi, nucleus, and cytoplasm [69]. As a membrane-bounded protein that associates with PD-1 for immunosuppression, PD-L1 must be properly transported from the synthesis site (ER) to the final destination (the plasma membrane) to ensure its physiological and cellular functions. In addition, PD-L1 homeostasis relies on recycling and degradation to balance the protein level. Protein trafficking through the secretory and endocytic pathways relies on membrane-bound vesicles and a complex set of proteins involved in vesicle formation, transport, docking, and fusion with the respective target membranes. The critical regulators of PD-L1 trafficking include HIP1R, exosomes, and ALIX [42,63,70], yet the exact mechanisms of PD-L1 transportation between different cell compartments remain unclear. There are many lingering questions about how PTMs of PD-L1 regulate their subcellular localization and thus contribute to the intracellular PD-L1 oncogenic function. First, is there a “trafficking code” that associates vesicle trafficking proteins, such as soluble N-ethylmaleimide-sensitive factor activating protein receptors (SNAREs) and small GTPases, which guide PD-L1′s intracellular journey? Second, how do PTMs regulate “the last mile” of the mature PD-L1 trafficking journey, from Golgi to the cell surface to reach PD-1 in another cell? Third, how do PTMs of PD-L1 impact the efficacy of PD-1/PD-L1-targeting immunotherapy? Most PD-L1 antibodies approved by the FDA are produced using synthetic peptide antigens or recombinant proteins expressed in *E. coli* or other host organisms, which do not harbor PTMs to recapitulate the native antigens in human cells. PTMs, especially glycosylation, could render their polypeptide antigens inaccessible to PD-L1 antibodies or affect PD-1–PD-L1 interaction. A better understanding of PD-L1 PTMs will help develop better diagnostic and therapeutic PD-L1 antibodies.

As the receptor of PD-L1, PD-1 is also subjected to PTMs, including ubiquitination, glycosylation, and fucosylation [71,72]. PD-1 expression is regulated by E3 ligases F-box protein 38 (FBXO38) [73], c-Cbl [74], and the Kelch-like protein 22–Cullin-3–Ring-box 1 (KLHL22–CUL3–RBX1) complex [75], which mediate K48-linked polyubiquitination and subsequent proteasome degradation. The KLHL22–CUL3–RBX1 complex also mediates the ubiquitination of incompletely glycosylated PD-1 and degradation of PD-1 before its transportation to the cell surface [75]. Fucosylation mediated by fucosyltransferase Fut8 at N49 and N74 regulates PD-1 cell surface expression. T cells treated with a cellular fucosylation inhibitor had a stronger antitumor reaction in vivo [76]. Loss of core fucosylation promotes FBXO38-mediated PD-1 ubiquitination and subsequent degradation by the proteasome [77]. Unlike PD-L1 glycosylation, which directly impacts PD-1–PD-L1 interaction, PD-1 glycosylation does not. The PD-1 glycosylation sites (N49, N58, and N116) are far away from the PD-1–PD-L1-binding interface [78]. Consistent with this information, the binding of two clinical anti-PD-1 antibodies (nivolumab and pembrolizumab) to PD-1 is not affected by PD-1 glycosylation [79,80]. Yet, N58 in PD-1 is required for the binding by two other anti-PD-1 antibodies (camrelizumab and MW11-h317) [81,82]. This reminds us that PD-1 glycosylation should be considered when designing PD-1-specific monoclonal antibodies for immune checkpoint therapy. Therefore, study on the mechanisms of PTMs of PD-L1 and PD-1 is needed for improving the efficacy of anti-PD-1/PD-L1 immunotherapy in the future.

In summary, a better understanding of PTMs in PD-1–PD-L1 interactions and regulation will pave the way for better immune checkpoint therapies.

## Figures and Tables

**Table 1:** PD-L1 modifications and their downstream impact.

Posttranslational Modification	Regulator	Downstream Impact	References
Glycosylation	STT3A	Transfers the core glycan structure to PD-L1, resulting in PD-L1 protein *N*-glycosylation and stabilization.	[27]
	B3GNT3	N192 and N200 of PD-L1 glycosylation are required for PD-L1–PD-1 interaction.	[37]
(De)Ubiquitination	β-Trcp	Promotes PD-L1 poly-ubiquitination and degradation following GSK3β-mediated T180 and S184 phosphorylation of PD-L1.	[24]
	Cullin-3-SPOP	Destabilization of PD-L1 through proteasomal degradation.	[30]
	STUB1	Poly-ubiquitinates and down-regulates PD-L1 through proteasomal degradation.	[53]
	CSN5	Deubiquitination of PD-L1 to enhance the stability of PD-L1.	[32]
	USP9X	Deubiquitinates and stabilizes PD-L1.	[33]
	USP22	Deubiquitinates and stabilizes PD-L1.	[34,35]
	OTUB1	Deubiquitinates and stabilizes PD-L1.	[36]
Phosphorylation	GSK3β	Phosphorylation of PD-L1 at T180 and S184 recruits b-TrCP for PD-L1 degradation.	[24]
	JAK1	Phosphorylation on Y112 enhances STT3A-mediated PD-L1 glycosylation and trafficking to the cell surface.	[28]
	AMPK	Phosphorylates PD-L1 at S195 to induce abnormal ER mannose trimming and promote PD-L1 degradation through the ERAD pathway.	[29]
Palmitoylation	ZDHHC9	PD-L1 palmitoylation at C272 maintains its protein stability and cell surface distribution.	[55]
	DHHC3	PD-L1 palmitoylation at C272 promotes PD-L1 storage and stabilization.	[56]
(De)Acetylation	HDAC2	PD-L1 is translocated into the nucleus and binds to DNA to regulate the expression of multiple immune-response-related genes.	[62]
	P300	Acetylated PD-L1 at the K263 site to maintain PD-L1 in cytoplasm.	[62]
